# Development and application of a rapid detection system for *Aspergillus fumigatus* based on ERA/CRISPR-Cas12a

**DOI:** 10.1186/s12866-026-04881-4

**Published:** 2026-03-09

**Authors:** Qiuyang Jiang, Xiaotong Zeng, Qi Zhang, Fo Yang, Tingyao Lv, Yushuo Zhang, Jin Wang, Feng Li, Dayong Xu

**Affiliations:** 1 Anhui Province Key Laboratory of Pollutant Sensitive Materials and Environmental Remediation, Huaibei Normal University, Huaibei, Anhui China; 2 School of Life Sciences, Huaibei Normal University, Huaibei, Anhui China; 3 The People’s Hospital, Huaibei, Anhui China; 4 Department of Clinical Laboratory, Shenzhen Institute of Translational Medicine, the First Affiliated Hospital of Shenzhen University, Shenzhen Second People’s Hospital, Shenzhen, Guangdong China

**Keywords:** Aspergillosis, ERA, CRISPR/Cas12a, One-Pot method, Lateral flow test strip

## Abstract

*Aspergillus fumigatus* (*AF*) is the predominant pathogen implicated in invasive aspergillosis (IA) in humans; therefore, prompt and accurate detection is critical for the effective prevention and management of IA. This study developed a rapid detection system targeting the *AF*-specific *anxC4* gene by integrating enzymatic recombinase amplification (ERA) with CRISPR/Cas12a. The reaction proceeds at a stable temperature of 37 °C, with amplification and detection systems separately positioned in the tube lid and bottom, respectively, effectively minimizing aerosol contamination typically associated with product transfers. To enhance sensitivity, the One-Pot method was optimized. Consequently, the fluorescence detection limit reached 1 fg/µL, and the sensitivity of the test strip reached 10 fg/µL, with no cross-reactivity observed against other fungi. Detection of *AF* was completed within 60 min, and results were visually displayed through fluorescence signals and nucleic acid test strips. Clinical practicality was further evaluated using aspergillosis samples, which demonstrated satisfactory performance. Pure culture results confirmed that out of 62 sputum samples, 32 were positive and 30 negative. Evaluation of 62 clinical samples using the One-Pot ERA-CRISPR/Cas12a system demonstrated sensitivity and specificity rates of 93.75% and 93.33%, respectively, via fluorescence detection, and 90.63% sensitivity and 96.67% specificity using lateral flow strips.

**Supplementary Information:**

The online version contains supplementary material available at 10.1186/s12866-026-04881-4.

## Introduction

Fungi are widely distributed in nature. Except for some superficial infections, severe fungal infections do not occur in humans with intact immune system. However, when the immune system is weakened, the risk of infection significantly increases. According to epidemiological data, fungal infections affect over one billion individuals worldwide [1]. Invasive fungal infections cause approximately 3.8 million deaths annually, exhibiting higher mortality rates compared to diseases such as tuberculosis and malaria [2, 3].

*AF* is a saprophytic filamentous fungus commonly found in soil and compost. It is also a pathogen causing various lung diseases in humans, birds, and other mammals [4]. It spreads via asexual spores and is the main pathogen responsible for IA. IA primarily affects individuals with compromised immune systems, including newborns who have immature immune defenses and older adults with age-related immune decline [5, 6]. Annually, over 2,113,000 individuals develop invasive aspergillosis associated with chronic obstructive pulmonary disease, intensive care, lung cancer, or hematological malignancies, resulting in approximately 1,801,000 deaths (85.2%). The annual incidence of chronic pulmonary aspergillosis is 1,837,272 cases, causing approximately 340,000 deaths (18.5%) [2] Without timely detection and treatment, mortality rates associated with IA are exceedingly high [7]. 

Currently, hospitals diagnose aspergillosis through fungal culture and microscopic examination. Although this diagnostic approach is highly specific, it requires substantial time. The Galactomannan test detects galactomannan, which is commonly identified in blood and bronchoalveolar lavage fluid (BALF) of suspected cases [8]. BALF detection has higher sensitivity than serum detection [9], but yields more false-positive results [10]. Molecular diagnostic approaches, such as polymerase chain reaction (PCR) and real-time quantitative PCR, exhibit excellent specificity. However, PCR techniques require specialized instruments and trained operators, restricting their practical application in resource-limited or remote healthcare settings [11]. Therefore, developing a rapid, accurate, and easily applicable detection method for *AF* in primary healthcare institutions holds significant clinical value.

Isothermal amplification techniques, including loop-mediated isothermal amplification (LAMP) [12, 13] and recombinase polymerase amplification (RPA) [14], are well-established for pathogen detection [4] due to their simplicity and instrument independence. Among these, enzymatic recombinase amplification (ERA) enables rapid amplification at constant temperature, ideal for point-of-care testing (POCT). The ERA technique utilized in this study is a commercialized and optimized system based on RPA principles. Similar to RPA, ERA employs recombinase enzymes to facilitate primer invasion into double-stranded DNA, followed by strand-displacement synthesis. The ERA kit offers proprietary, pre-formulated, lyophilized reagents that enhance stability and user-friendliness. The innovation of this work is our One-Pot"lid-bottom separation” strategy, which integrates ERA with CRISPR/Cas12a. This design physically separates amplification and detection, effectively preventing contamination while maintaining high sensitivity.

The CRISPR/Cas system is widely utilized in molecular diagnostics due to its highly specific target recognition. Guided by crRNA, Cas12a recognizes target DNA and activates non-specific cleavage, indiscriminately cutting surrounding single-stranded DNA reporter molecules [15]. This feature makes CRISPR/Cas12a an ideal tool for highly sensitive fluorescence or lateral flow detection. Li et al. [16] first combined CRISPR/Cas12a with PCR, developing a rapid and sensitive detection method termed One-Hour Low Cost Multipurpose Gene Expert System (HOLMES). Subsequently, Chen et al. [15] established DETECTR, a CRISPR/Cas12a detection platform based on RPA, demonstrating sensitivity at the attomolar level and extremely high specificity. Biosensing systems based on CRISPR/Cas12a have subsequently been developed for detecting diverse targets, including pathogenic bacteria [17, 18], genetically modified crops [19, 20], SARS-CoV-2 [21, 22], and other pathogenic microorganisms [23–27]. Integrating CRISPR/Cas12a with isothermal amplification significantly increases target abundance quickly, thus greatly enhancing detection sensitivity.

In this study, the ERA-CRISPR/Cas12a system was applied for rapid AF detection by selecting the *anxC4* gene as the diagnostic target. This gene encodes a novel annexin specific to filamentous fungi, differing significantly in sequence from conventional annexins and other fungal genes [28]. This difference facilitates the design of highly specific oligonucleotides. Highly specific primers and crRNA targeting the conserved *anxC*4 gene of *AF* were designed and integrated with lateral flow test strips. Moreover, by separating the reaction and detection components into distinct locations within a single centrifuge tube, this optimized One-Pot configuration effectively mitigated common issues such as aerosol contamination and low amplification efficiency inherent in traditional methods. The reliability of this method was verified using clinical samples. The established ERA-CRISPR/Cas12a technique offers a novel diagnostic approach for early *AF* infection detection and presents a valuable reference for rapid identification of other fungal pathogens.

## Materials and methods

### Design of specific primers and crRNA

**Fig. 1:**
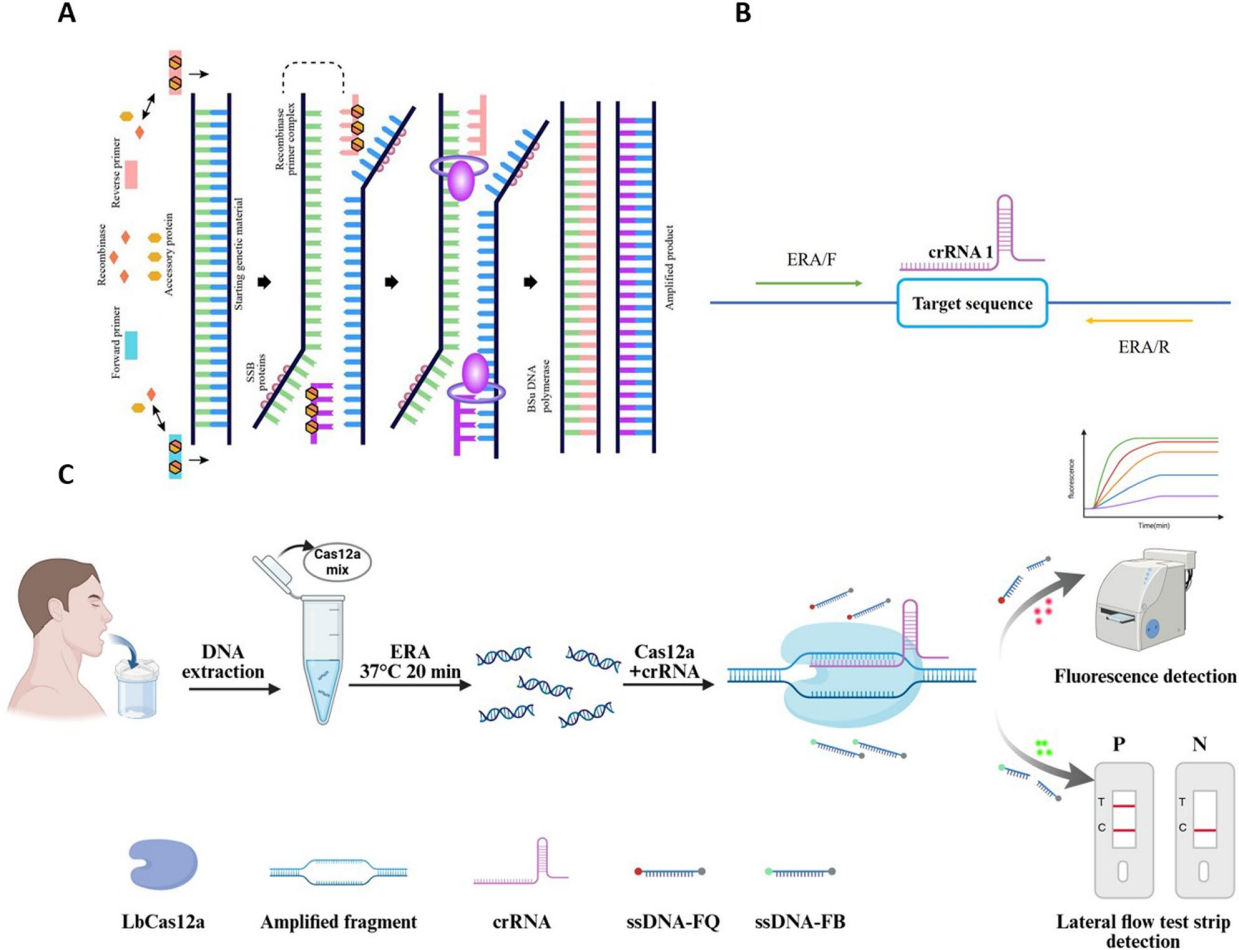
Schematic diagram of CRISPR-Cas12a coupled with ERA for fluorescent and lateral flow detection. **A** Schematic diagram of the ERA amplification principle. **B** Schematic diagram of crRNA binding to the target sequence. **C** The workflow of the One-Pot ERA-CRISPR/Cas12a system for detection of *Aspergillus fumigatus*

The *anxC4* gene of *AF* (GenBank accession AY598940) was selected. Using the online tool CRISPR RGEN, two high-scoring crRNA sequences (41 nt) targeting a specific amplification fragment of the *anxC4* gene were designed. crRNA sequences (crRNA 1 and crRNA 2) were synthesized using the Cas12a High-Yield crRNA Synthesis and Purification Kit. One set of PCR primers (PCR-F/R) and three pairs of ERA primers (ERA-F1/R1, ERA-F2/R2, and ERA-F3/R3) were specifically designed within target regions of the crRNA sequences using Primer Premier 5 and validated via NCBI BLAST. The schematic diagram of the ERA amplification principle is shown in Fig. 1A, and the schematic diagram of crRNA binding to the target sequence is shown in Fig. 1B. All the primers used are listed in Table 1.

### CRISPR-Cas12a detection system

The CRISPR-Cas12a detection reaction (20 µL) contained 2 µL of 10× HOLMES Buffer, 0.5 µL of Cas12a(10 µM), 2 µL of crRNA (3 µM), 2 µL of ssDNA (3 µM) reporter probe, and nuclease-free water up to a final volume of 20 µL. Fluorescence measurements were conducted using the Roche LightCycler^®^ 96 at 37 °C, recording fluorescence signals every 30 s over 45 min (90 cycles).

**Table 1:** Primers and crRNA used in this study

Name	Sequence (5′-3′)	Length	Reference
PCR/F	CCTCTGCGAACAACCTTT	18	This study
PCR/R	GCTCTTTGCGATCCCTTT	18	This study
ERA/F1	CCAGTCATTCTGTCTCCTCTGCGAACAACC	30	This study
ERA/R1	CTTCAGCTCTTTGCGATCCCTTTCATCCTT	30	This study
ERA/F2	TCTGTCTCCTCTGCGAACAACCTTTCAGTC	30	This study
ERA/R2	CTTCAGCTCTTTGCGATCCCTTTCATCCTT	30	This study
ERA/F3	TCATTCTGTCTCCTCTGCGAACAACCTTTC	30	This study
ERA/R3	CTTCAGCTCTTTGCGATCCCTTTCATCCTT	30	This study
crRNA 1	UAAUUUCUACUAAGUGUAGAUGCGAGUGUUUCCUCCUCCCG	41	This study
crRNA 2	UAAUUUCUACUAAGUGUAGAUAUCCUUGGACUUGUUUCGCG	41	This study

### ERA primers and crRNA screening

Three pairs of ERA primers were tested for amplifying *AF* genomic DNA, selecting the primer set yielding a single specific amplification product. Two crRNAs were individually assessed within the CRISPR/Cas12a system. Optimal ERA primer and crRNA combinations were selected by evaluating endpoint fluorescence intensities and amplification curve profiles. Nuclease-free water served as a negative control and genomic DNA extracted from the *AF* reference strain was used as the standard positive. in all experiments. Each experimental condition was independently replicated three times.

### Establishment and optimization of two-step CRISPR/Cas12a detection system

The established CRISPR/Cas12a assay incorporated two detection approaches: fluorescence-based detection and lateral flow assay (LFA). Fluorescence detection was conducted at 37 °C using the quantitative PCR system, whereas lateral flow detection employed incubation at 37 °C in a water bath. For LFA, reaction samples were diluted to a total volume of 50 µL using nuclease-free water, thoroughly mixed, and test strips were immersed in the solution to visually assess the results at the test line.

Optimization of Fluorescence Detection System: With DNA template concentration fixed at 1 nM, Cas12a/crRNA ratios (2.5:1, 1.25:1, 1:1, 1:1.2, 1:1.6, and 1:2) and ssDNA probe concentrations (50, 100, 200, 300, 400, and 500 nM) were optimized. Nuclease-free water was the negative control. Fluorescence intensities were recorded under each condition.

Optimization of Test Strip Detection System: ssDNA probe concentrations were diluted (0, 100, 200, 300, 400, 600, and 800 nM) and tested. The lowest concentration causing T-line disappearance was selected as optimal.

Optimization of CRISPR/Cas12a Reaction Time: Reaction times (5, 10, 15, 20, 25, and 30 min) were evaluated for both fluorescence and strip detection. Optimal time was determined based on T-line color intensity.

### Sensitivity evaluation of the two-step CRISPR/Cas12a detection system

Sensitivity of PCR-CRISPR/Cas12a and ERA-CRISPR/Cas12a methods was compared. DNA templates were serially diluted (10^6^ to 10^0^aM) and subjected to PCR and ERA amplification, respectively. Amplification products (2 µL) were analyzed using fluorescence detection, and fluorescence curves were recorded. Genomic DNA was diluted (100 pg/µL to 0.1 fg/µL) to further validate the detection performance.

### Establishment of the One-Pot ERA-CRISPR/Cas12a detection system

**Fig. 2:**
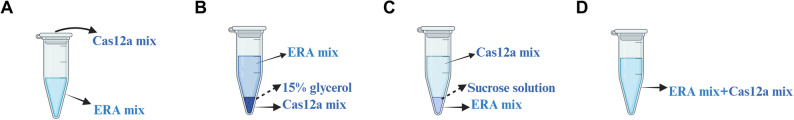
Four strategies to achieve the One-Pot method by combining ERA with CRISPR/Cas12a. **A** The ERA mixture product is placed at the bottom of the tube, and the Cas12a mixture is placed on the lid. **B** The Cas12a detection system mixed with 15% glycerol is placed at the bottom of the tube, with the ERA mixture added on top. **C** The ERA mixture, thoroughly mixed with sucrose, is placed at the tube bottom, and the Cas12a system is placed above. **D** The ERA and Cas12a mixtures are simultaneously combined at the bottom of the tube

The workflow of the One-Pot ERA-CRISPR/Cas12a system for detection of *Aspergillus fumigatus* (Fig. 1C). The ERA reaction mixture comprised 20 µL of dissolving agent, 20 µL of nuclease-free water, and forward and reverse primers (2.5 µL each, 10 µM), ensuring complete dissolution of the lyophilized reagent powder. The Cas12a detection solution contained 0.5 µL LbCas12a (10 µM), 2 µL crRNA (3 µM), 2 µL 10× HOLMES Buffer, and 2 µL ssDNA reporter probe (3 µM). Before initiating the reaction, 0.4 µL of template DNA (100 pg/µL) was combined with 1 µL of activator, forming the final sample mixture. Four variants of the One-Pot detection protocol were assessed: (1) Initially, 8.6 µL ERA mixture was placed at the bottom of a centrifuge tube, followed by adding 10 µL Cas12a detection mixture onto the lid. Subsequently, 1.4 µL of the sample mixture was rapidly transferred into the ERA mixture, and the tube was immediately sealed and incubated at 37 °C for 20 min. After incubation, gentle rotation mixed the solutions, and fluorescence analysis was performed using a fluorescence quantitative PCR system (Fig. 2A). (2) 15% glycerol was added between the ERA and Cas12a mixtures (Fig. 2B). (3) 1.765 mg sucrose was added to the ERA mixture (Fig. 2C). (4) ERA and Cas12a mixtures were directly combined before simultaneous amplification and detection (Fig. 2D).

### Optimization of the One-Pot ERA-CRISPR/Cas12a detection system

The volume of genomic DNA (100 pg/µL) used in the One-Pot ERA system was increased to 1 µL. The final concentration of the ssDNA FB probe was fixed at 300 nM, while the Cas12a protein concentration was optimized at 125, 250, 500, 750, and 1000 nM. The ERA reaction times tested were 5, 10, 15, 20, 25, 30, and 60 min, and detection time was set at 30 min.

### Sensitivity evaluation of the One-Pot ERA-CRISPR/Cas12a detection system

To determine the sensitivity of the established One-Pot ERA-CRISPR/Cas12a assay for *AF* detection, serial 10-fold dilutions of genomic DNA were prepared and analyzed. Negative controls utilized nuclease-free water. Sensitivity was evaluated based on the intensity of fluorescence signals and visual presence of the T-line on lateral flow strips.

### Specificity evaluation of the One-Pot ERA-CRISPR/Cas12a detection system

Specificity was assessed by testing genomic DNA extracted from all strains listed in Table 2, using the optimized One-Pot ERA-CRISPR/Cas12a system. Nuclease-free water served as a negative control. Fluorescence signal intensities and test-strip T-line visualization were used to evaluate the specificity. In addition to the fungal strains listed in Table 2, an expanded panel (Supplementary Table S2) was tested to rigorously evaluate both the inclusivity and exclusivity of the assay.

**Table 2:** Fungal strains used in this study

Strain	Source of the strain	Strain quantity
*Aspergillus fumigatus*	ATCC MYA-4609	1
*Candida albicans*	ATCC 5314	1
*Candida tropicalis*	ATCC 13,803	1
*Aspergillus niger*	CICC 40,102	1
*Penicillium chrysogenum*	CGMCC 3.15725	1
*Alternaria alternata*	CICC 40,758	1
*Botrytis cinerea*	CGMCC 3.3790	1
*Aspergillus cristatus*	CICC 41,784	1

### Clinical sample processing and DNA extraction

Sputum samples collected from individuals under investigation for aspergillosis were cryopreserved at -80 °C until nucleic acid extraction. The processing protocol commenced with sputum liquefaction using a digestive solution, followed by vortexing for 15–30 min to achieve complete homogenization. A 500 µL aliquot of the resulting homogenate was used for genomic DNA extraction with a commercial kit, performed strictly according to the manufacturer’s instructions, and the extracted DNA was eluted in 50 µL of the provided buffer. DNA purity and concentration were assessed spectrophotometrically. Samples with an A260/A280 ratio between 1.8 and 2.0 were deemed to have acceptable purity and were subsequently subjected to downstream analyses.

### Clinical evaluation of the One-Pot ERA-CRISPR/Cas12a detection system

Sixty-two sputum samples (32 positives diagnosed with *AF*, 30 negatives without *AF*) were collected from Huaibei People’s Hospital (approved by the hospital ethics committee). These clinical samples underwent diagnosis by pure culture, qPCR, and the One-Pot ERA-CRISPR/Cas12a detection method.

## Results

### Screening of ERA primers, PCR primers, and crRNA

**Fig. 3:**
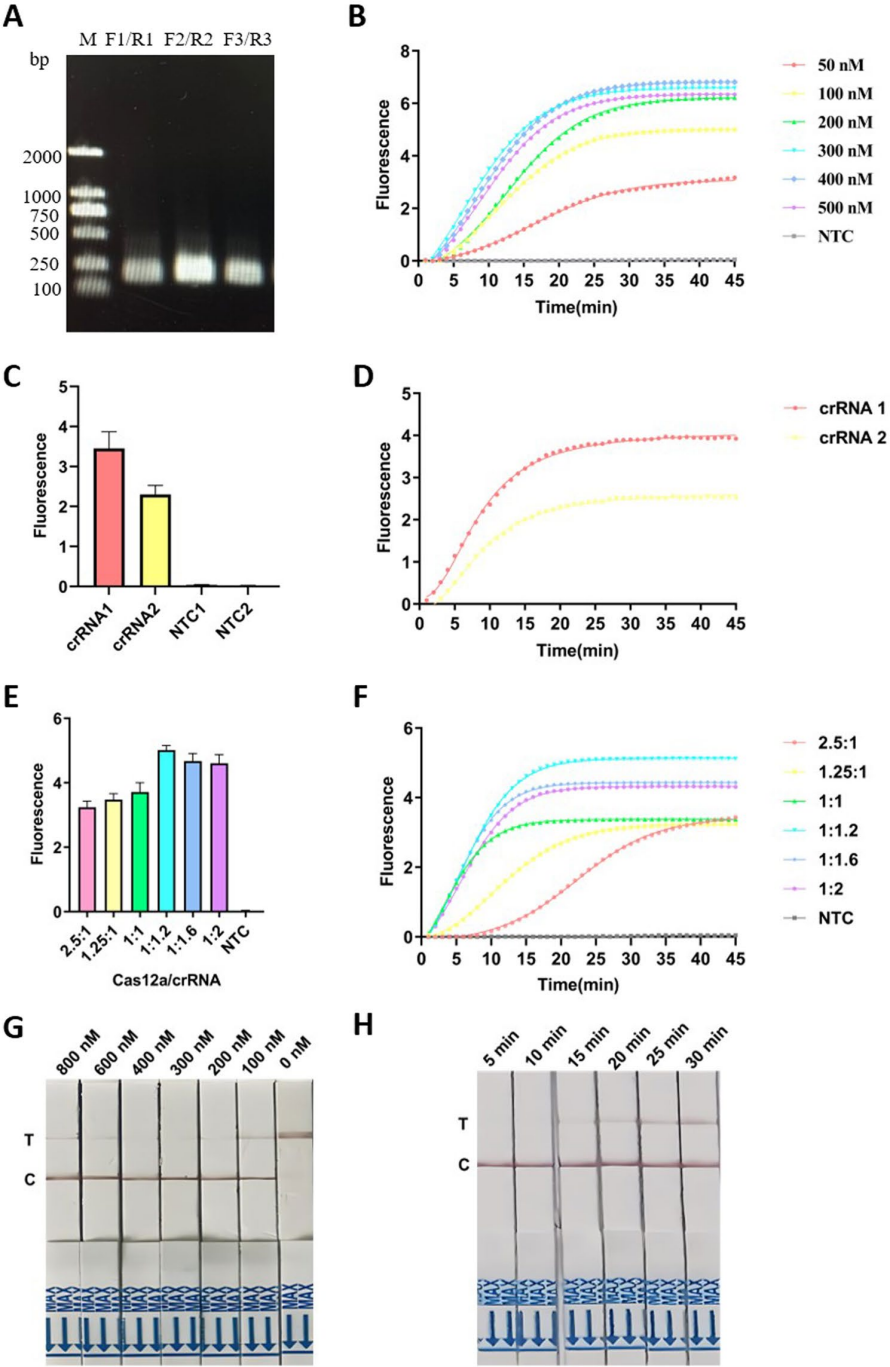
Optimization of the ERA/CRISPR-Cas12a fluorescence system for detecting *AF*. **A** Screening of ERA primers for amplification of *AF*, with gel electrophoresis analysis. M: 2000 bp DNA Marker. **B** Optimization of ssDNA FQ concentration, showing fluorescence curves at different concentrations. **C** Fluorescence intensities of different crRNAs after 45 min. **D** Screening of optimal crRNA using real-time fluorescence curves. **E** Fluorescence intensities of different Cas12a/crRNA ratios after 45 min. **F** Optimization of ERA/CRISPR-Cas12a reaction, comparing fluorescence curves at various Cas12a/crRNA ratios. **G** Optimization of lateral flow strip detection by evaluating the effects of ssDNA FB concentrations on the detection system without target DNA. T represents the test line, and C represents the control line. **H** Effect of different CRISPR/Cas12a incubation times on test strip T-line visibility

ERA was performed using 1 nM genomic DNA of *AF* and three primer sets, followed by electrophoresis. Although all ERA primer sets produced a single band, ERA primer set 2 yielded a brighter amplification band (Fig. 3A). Optimal primer selection was performed according to band intensity from electrophoresis results, followed by crRNA screening. Fluorescence curves for crRNA1 and crRNA2 revealed that crRNA1 exhibited superior performance (Fig. 3C, D). Although both crRNAs reached plateau signals after approximately 25 min, crRNA1 demonstrated approximately 1.5-fold greater fluorescence intensity than crRNA2. Thus, primer set ERA/F2, R2 (252 bp) and crRNA1 were selected as the optimal combination for subsequent experiments.

### Establishment of the two-step ERA-CRISPR/Cas12a detection system

To optimize the Cas12a/crRNA reaction, six concentration ratios were tested. Results demonstrated maximum fluorescence accumulation at the Cas12a/crRNA ratio of 1:1.2 (Fig. 3E, F).

The fluorescence detection reporter molecule (ssDNA FQ) concentration was further optimized (Fig. 3B). Fluorescence intensity increased steadily from 50 nM up to 300 nM and reached saturation thereafter. Considering both detection cost and experimental effectiveness, 300 nM was selected as the optimal concentration. At this point, a Cas12a concentration of 250 nM and a crRNA concentration of 300 nM were selected as optimal.

Optimization of the lateral flow test strip detection system (ssDNA FB, Fig. 3G) showed that lower concentrations resulted in false-positive T-line bands. As ssDNA FB concentration increased, T-line band intensity decreased while C-line intensity increased.

At concentrations of 300 nM and 400 nM, false-positive signals were eliminated. Considering these results, 300 nM ssDNA FB was chosen for subsequent strip-based detection. The test strip shows two visible red lines 15 min after the test, and clear C-line and T-line can be observed at 30 min. Therefore, the detection time for all subsequent test strips is set to 30 min (Fig. 3H).

**Fig. 4:**
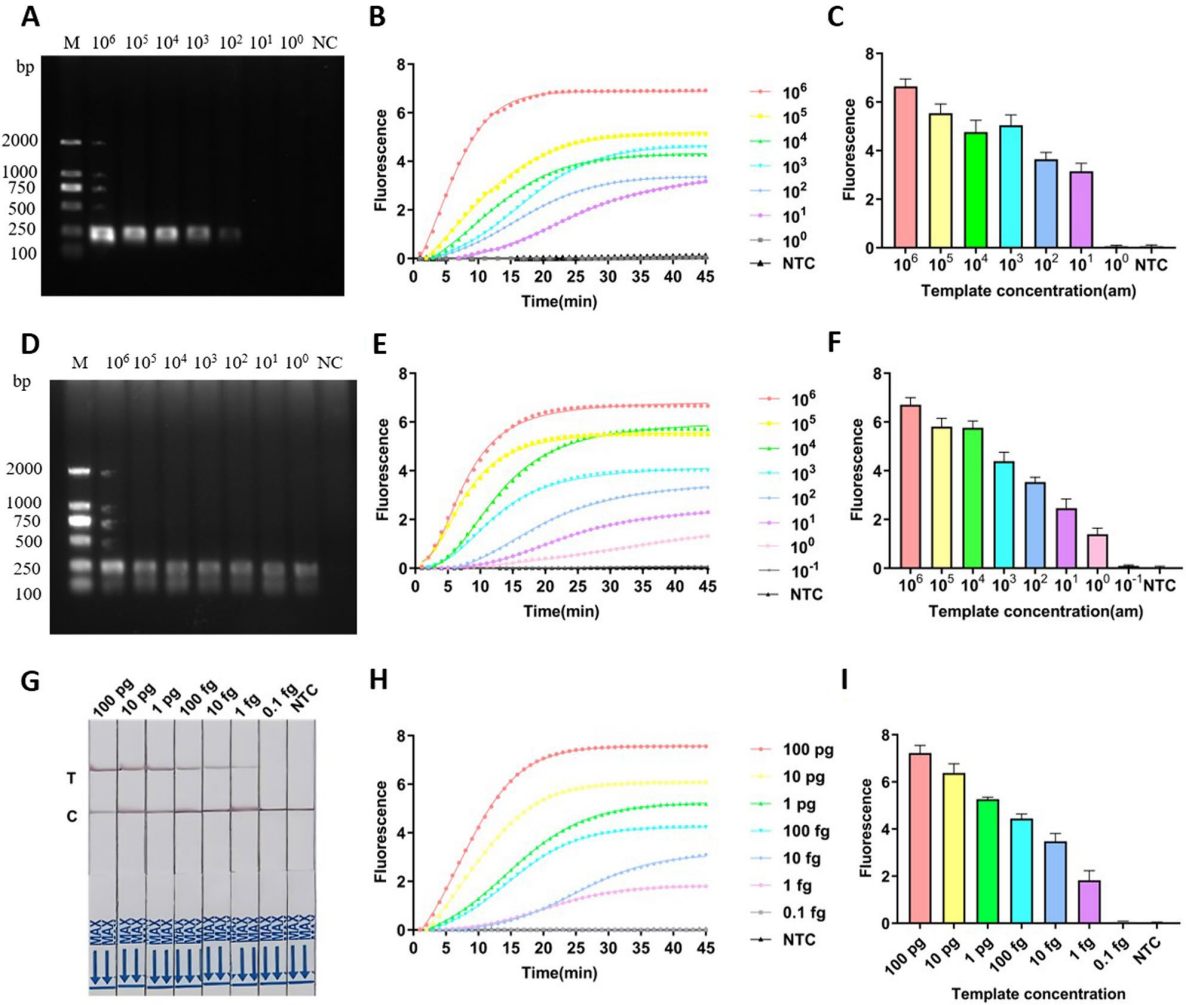
Sensitivity analysis of Two-Step CRISPR/Cas12a fluorescence detection. **A**-**C** 1% agarose gel electrophoresis of PCR products amplified from 10^6^–10^0^ aM DNA template, CRISPR/Cas12a fluorescence detection curves of PCR products at different concentrations, and fluorescence intensities at the end of detection. **D**-**F** Gel electrophoresis, fluorescence detection curves, and final fluorescence intensities of ERA products at various concentrations. **G** Sensitivity of lateral flow assay (LFA) detection using genomic DNA ERA products (100 pg–0.1 fg). **H**, **I** Fluorescence detection curves and fluorescence intensities of ERA products amplified from genomic DNA at concentrations ranging from 100 pg to 0.1 fg using CRISPR/Cas12a

### Sensitivity test of the two-step ERA-CRISPR/Cas12a system

The sensitivity of the Two-Step ERA-CRISPR/Cas12a detection method was compared to that of the Two-Step PCR-CRISPR/Cas12a method. *AF* DNA was diluted from 10^6^ to 10^0^ aM. PCR alone did not produce visible amplification bands below 10^2^ aM (Fig. 4A). When PCR was combined with CRISPR/Cas12a detection, sensitivity improved to 10^1^ aM (Fig. 4B, C). ERA amplification products were visible by electrophoresis at 10^0^ aM (Fig. 4D). However, fluorescence detection using ERA-CRISPR/Cas12a was not successful below 10^0^ aM, setting its detection limit at 10^0^ aM (Fig. 4E, F).

To further evaluate sensitivity, extracted genomic DNA was serially diluted (100 pg/µL to 0.1 fg/µL). At high DNA concentrations, the ERA-CRISPR/Cas12a fluorescence reaction proceeded rapidly, reaching saturation within 20 min. Lower concentrations reacted more slowly, did not reach saturation within 45 min, and generated lower fluorescence values. Nevertheless, even at low concentrations, fluorescence signals were significantly stronger than those of the negative control. The detection limit of this method reached 1 fg/µL, indicating excellent sensitivity (Fig. 4H, I). The sensitivity of the Two-Step ERA-CRISPR/Cas12a method developed here exceeded that of conventional PCR-CRISPR/Cas12a. ERA was rapid and required no temperature cycling, making it more suitable for lateral flow strip visualization. The detection limit using lateral flow test strips matched the fluorescence detection method, both detecting down to 1 fg/µL (Fig. 4G).

### Establishment and optimization of the One-Pot ERA-CRISPR/Cas12a detection system

**Fig. 5:**
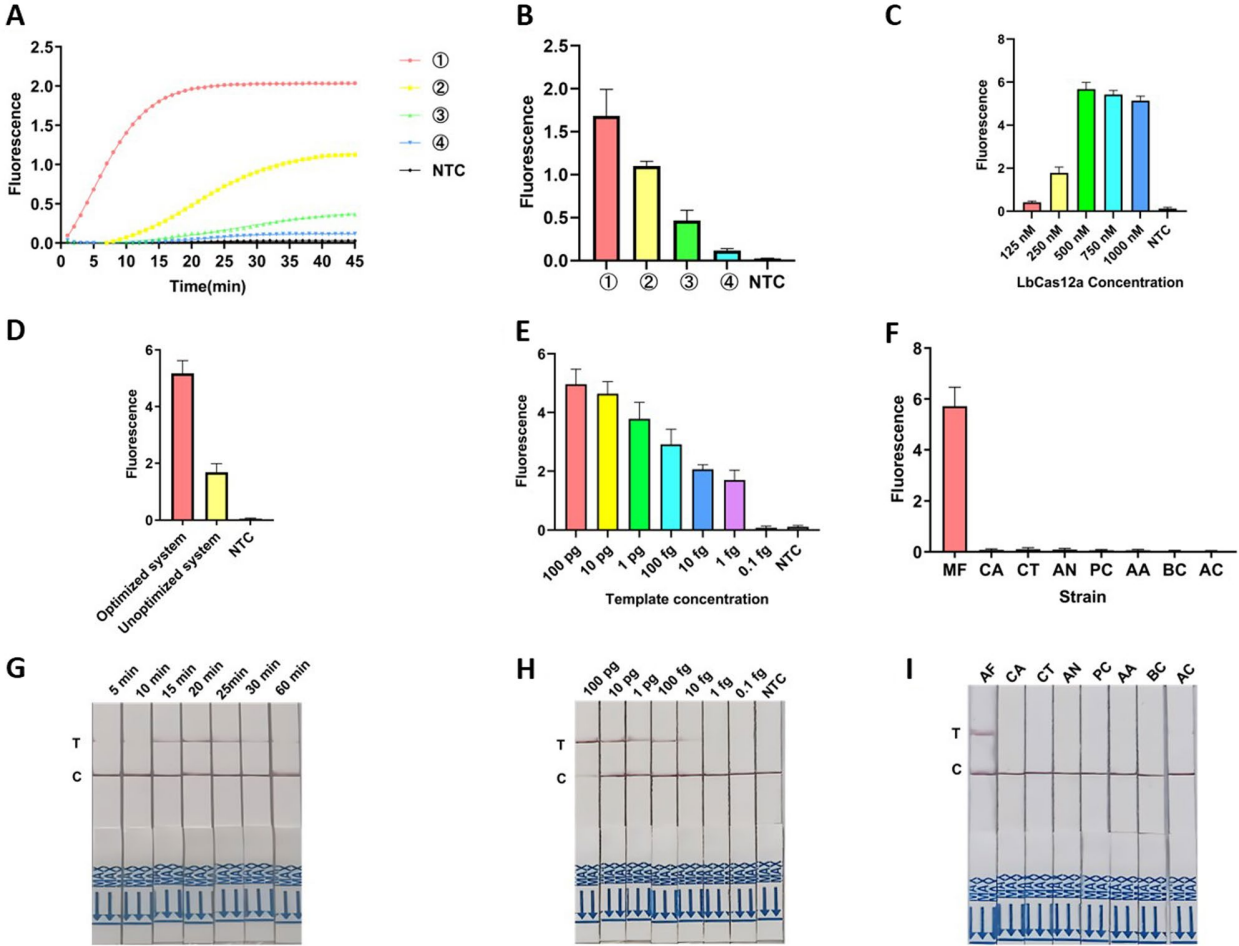
Establishment, optimization and application of the One-Pot ERA-CRISPR/Cas12a detection system. **A**, **B** Establishment of One-Pot method: ① Fluorescence curves and values of tube-cap separation method; ②Fluorescence curves and values with glycerol added to the Cas12a system; ③Fluorescence curves and values with sucrose added to the Cas12a system; ④Fluorescence curves and values of simultaneous amplification and detection. **C** Optimization of LbCas12a concentration for the One-Pot method. **D** Fluorescence curves of optimized One-Pot detection. **E**, **H** Sensitivity evaluation of the One-Pot ERA-CRISPR/Cas12a system using fluorescence and strip detection with genomic DNA concentrations from 100 pg to 0.1 fg. **F**, **I** Specificity analysis of the ERA/CRISPR-Cas12a fluorescence and strip detection systems: AF, *Candida albicans* (CA), *Candida tropicalis* (CT), *A. niger* (AN), *Penicillium chrysogenum* (PC), *Alternaria alternata* (AA), *Botrytis cinerea* (BC), *Aspergillus cristatus* (AC). **G** Optimization of amplification incubation times for One-Pot ERA detection

In the Two-Step ERA-CRISPR/Cas12a method, ERA and Cas12a detection occur separately, requiring transfer of amplification products. Due to the high amplification efficiency of ERA, aerosol contamination can easily occur during transfer, leading to false-positive results. Direct mixing of the ERA and Cas12a systems for fluorescence detection significantly reduced sensitivity and fluorescence intensity (Fig. 5A, B). Given that ERA amplification and Cas12a cleavage utilize the same DNA substrate, directly combining both reactions simultaneously in a single container negatively impacts assay sensitivity. To mitigate this, the ERA amplification reagents were initially placed at the bottom of the reaction tube, while the Cas12a reaction components were added separately onto the tube lid. Upon completion of ERA, the Cas12a mixture was centrifuged into the ERA solution, enabling subsequent reaction and detection. This approach generated clear fluorescence curves. Although glycerol and sucrose slightly improved sensitivity, their effects were less pronounced than the tube-bottom and lid separation method. Furthermore, glycerol and sucrose increased solution viscosity, hindering test strip detection and visualization. 

After comparing various One-Pot strategies, the method of placing ERA at the tube bottom and Cas12a at the lid was chosen. Following ERA, centrifugation mixed the two solutions, eliminating aerosol contamination and avoiding substrate competition, thereby enhancing sensitivity. To further optimize sensitivity, component ratios were adjusted. The optimal Cas12a concentration was determined to be 500 nM after increasing the template volume to 1 µL at 100 pg/µL (Fig. 5C). The optimized One-Pot assay achieved a sensitivity comparable to that of the Two-Step method (Fig. 5D), with a detection limit of 1 fg/µL by fluorescence detection (Fig. 5E). Under identical conditions, lateral flow test strips exhibited a slightly reduced sensitivity of 10 fg/µL (Fig. 5H), one order of magnitude lower than fluorescence detection. Amplification time optimization indicated that two distinct red lines appeared at 15 min. However, after amplification exceeded 25 min, the T-line signal weakened significantly, indicating that excessively long amplification times reduced test effectiveness (Fig. 5G).

### Specificity evaluation of the One-Pot ERA-CRISPR/Cas12a detection system

Genomic DNA of all strains listed in Table 2 was used to evaluate specificity. Results showed (Fig. 5F, I) that only the *AF* samples generated significant fluorescence signals and two clear red bands on test strips, indicating positive results. Furthermore, an expanded panel—including additional clinical isolates of *AF*, phylogenetically closely related fungal species, and other common fungal pathogens was evaluated (Supplementary Table S2). All non-target species generated either no detectable fluorescence signal or solely the control line (C-line) on the lateral flow test strips (Supplementary Fig. S2). Thus, this method demonstrated excellent specificity.

**Table 3:** Comparison of detection results between the One-Pot ERA-CRISPR/Cas12a fluorescence system and qPCR

		qPCR			Sensitivity	Specificity	Consistency
		Positive	Negative	Total			
One-Pot ERA-CRISPR/Cas12aFluorescence detection system	Positive	30	2	32			
	Negative	2	28	30	93.75%	93.33%	93.55%
	Total	32	30	62			

Sensitivity = 93.75% (95% CI: 80.6%-98.3%); Specificity = 93.33% (95% CI: 79.7%-98.2%); kappa = 0.87

**Table 4:** Comparison of detection results between the One-Pot ERA-CRISPR/Cas12a test strip detection system and qPCR

		qPCR			Sensitivity	Specificity	Consistency
		Positive	Negative	Total			
One-Pot ERA-CRISPR/Cas12a test strip detection system	Positive	29	1	30			
	Negative	3	29	32	90.63%	96.67%	93.55%
	Total	32	30	62			

Sensitivity = 90.63% (95% CI: 76.2%-96.7%); Specificity = 96.67% (95% CI: 83.3%-99.9%); kappa = 0.87

### Clinical evaluation of the One-Pot ERA-CRISPR/Cas12a system

**Fig. 6:**
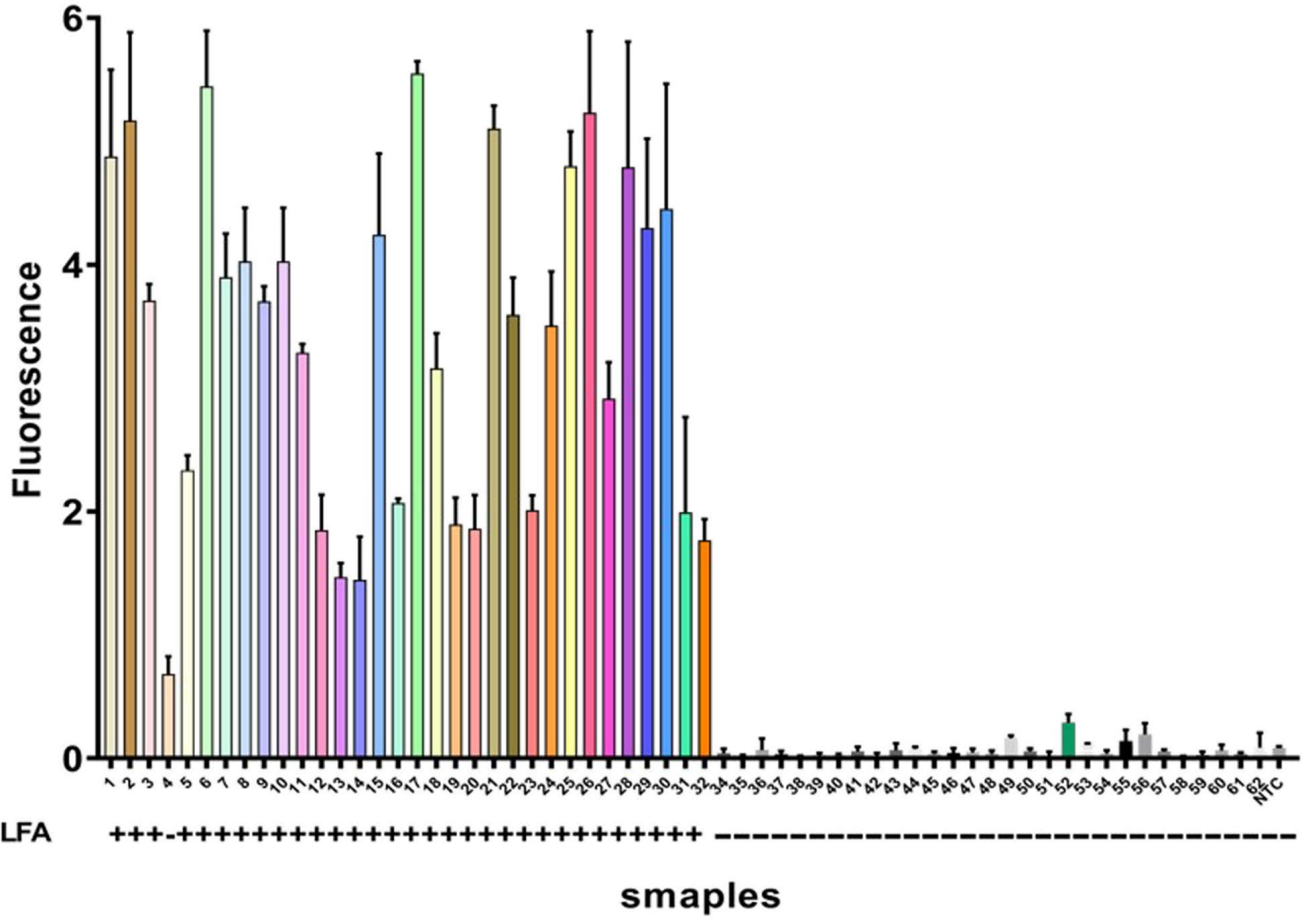
Clinical sample testing results: Samples 1–32 are positive, and samples 33–62 are negative clinical samples. LFA: “+” indicates positive results, “–” indicates negative results

Sixty-two sputum samples were tested using traditional culture, One-Pot ERA-CRISPR/Cas12a, and fluorescence quantitative PCR methods. Results showed 32 positive and 30 negative samples. Overall, results obtained by the One-Pot ERA-CRISPR/Cas12a method aligned closely with culture-based findings (Fig. 6). Compared to qPCR, fluorescence-based ERA-CRISPR/Cas12a demonstrated a sensitivity of 93.75% and specificity of 93.33% (Table 3), while lateral flow strip assays showed a sensitivity of 90.63% and specificity of 96.67% (Table 4). The One-Pot ERA-CRISPR/Cas12a platform demonstrated high diagnostic concordance with quantitative PCR (qPCR) across both detection modalities. The fluorescence-based assay achieved a sensitivity of 93.75% (95% CI: 80.6–98.3%) and a specificity of 93.33% (95% CI: 79.7%–98.2%), while the lateral flow strip readout showed a sensitivity of 90.63% (95% CI: 76.2%–96.7%) and a specificity of 96.67% (95% CI: 83.3%–99.9%). Both formats exhibited excellent agreement with qPCR, yielding a Cohen’s kappa coefficient of 0.87 (95% CI: 0.75–0.99) for each. These findings demonstrate that the developed system provides robust and reliable diagnostic performance, adaptable to diverse testing settings.

## Discussion

Unlike traditional PCR, the ERA approach amplifies nucleic acids isothermally, eliminating the need for complex thermal cycling and simplifying operation. The ERA-CRISPR/Cas12a system developed in this study demonstrates a ten-fold improvement in analytical sensitivity compared to PCR-CRISPR/Cas12a assays, attributable to enhanced amplification efficiency under isothermal conditions. While fluorescence detection offers excellent quantitative capability, its reliance on specialized instrumentation limits applicability in resource-constrained settings. To overcome this limitation, lateral flow test strips replace fluorescence probes, allowing visual interpretation of results and enabling rapid, on-site detection without sophisticated equipment [29].

A fundamental challenge in One-Pot nucleic acid detection is preventing premature Cas12a activation during amplification. The addition of glycerol or sucrose increases the viscosity of the reaction system, reducing molecular diffusion. This approach prevents premature interaction of Cas12a with amplification products, avoiding inhibition of amplification [29, 30]. Although the addition of glycerol and sucrose can enhance detection sensitivity, the Cas12a enzyme may exhibit slow cleavage activity toward the target DNA during the amplification process, thereby reducing the availability of templates for subsequent amplification steps. This issue is particularly pronounced in samples with low concentrations, ultimately compromising the overall sensitivity of the reaction. In contrast, the bottom-separated approach ensures complete physical separation between the amplification system and the CRISPR detection system, preventing any premature interaction with Cas12a prior to the completion of amplification. This effectively avoids the degradation of target DNA or primers, resulting in a more reliable and sensitive detection performance compared to the use of glycerol and sucrose in One-Pot assays.

Compared to previously reported RPA-CRISPR/Cas12a [31]or RPA-CRISPR/Cas13a platforms [32], our system offers distinct operational and performance advantages. The 37 °C isothermal protocol reduces equipment complexity, while lyophilized reagent formulations improve stability for field applications. With detection limits of 1 fg/µL (fluorescence) and 10 fg/µL (LFA), the assay achieves sensitivity sufficient for clinical use. Clinical validation using 62 sputum samples showed strong concordance with qPCR (kappa = 0.87), confirming diagnostic reliability in practical clinical environments. When compared with traditional methods, our approach delivers substantially faster results (55 min) than culture (3–7 days), while maintaining sensitivity comparable to qPCR but with significantly reduced infrastructure requirements.

The assay also has several limitations that should be considered. Primarily, it is designed to specifically detect *AF* by targeting the *anxC4* gene. Bioinformatics analysis indicates that the primers used in this method can specifically target *A. fumigatus* and may also detect its closely related species, *A. lentulus* (Supplementary Table S1). From a clinical diagnostic standpoint, this cross-detection holds potential value, as *A. lentulus* belongs to the *Aspergillus fumigatus* complex and is itself an emerging pathogen associated with IA. However, other *Aspergillus* species, such as *A. flavus*、*A. terreus* and *A. clavatus*, are also recognized as clinically significant pathogens and are not identified by this method (Supplementary Fig. S1), future work should focus on developing a multiplex platform capable of simultaneously detecting multiple species. Additionally, the current assay relies on genomic DNA extraction from samples, resulting in a procedural disconnect between the extraction step and downstream detection. The current design requires a centrifugal step to integrate the amplification and detection modules for spatial separation. This requirement for laboratory infrastructure hinders the development of a fully instrument-free operational workflow. Future development should focus on integrating sample preparation into a closed, integrated device to facilitate deployment in settings with limited resources. In summary, the One-Pot ERA-CRISPR/Cas12a assay described here combines high sensitivity, operational simplicity, and visual readout capability, representing a promising tool for the rapid detection of *AF* in both clinical and resource-constrained environments. Further development toward multiplex detection and full workflow integration will expand its diagnostic usefulness and potential for clinical application.

## Supplementary Information

Supplementary Material 1.

Supplementary Material 2.

Supplementary Material 3.

Supplementary Material 4.

Supplementary Material 5.

## Data Availability

All data generated or analyzed herein are included in this manuscript.
