# Supplementary figures and images for: Development and application of a rapid detection system for Aspergillus fumigatus based on ERA/CRISPR-Cas12a
Source: BMC Microbiol. 2026 Mar 9;26:359. doi: 10.1186/s12866-026-04881-4 (PMC13085300; doi:10.1186/s12866-026-04881-4)

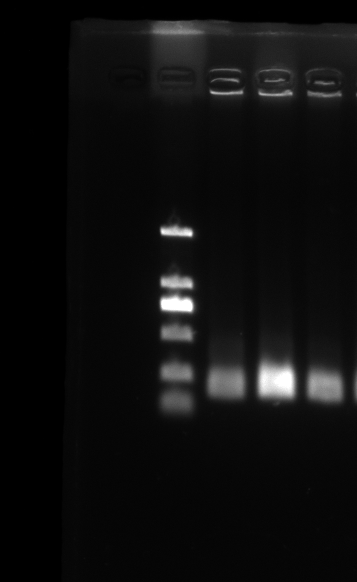

Supplement: Supplementary file 1 — Supplementary Material 1. [file 12866_2026_4881_MOESM1_ESM.zip › Supplementary materials - Original glue drawing/Original image(Fig. 2A).png]

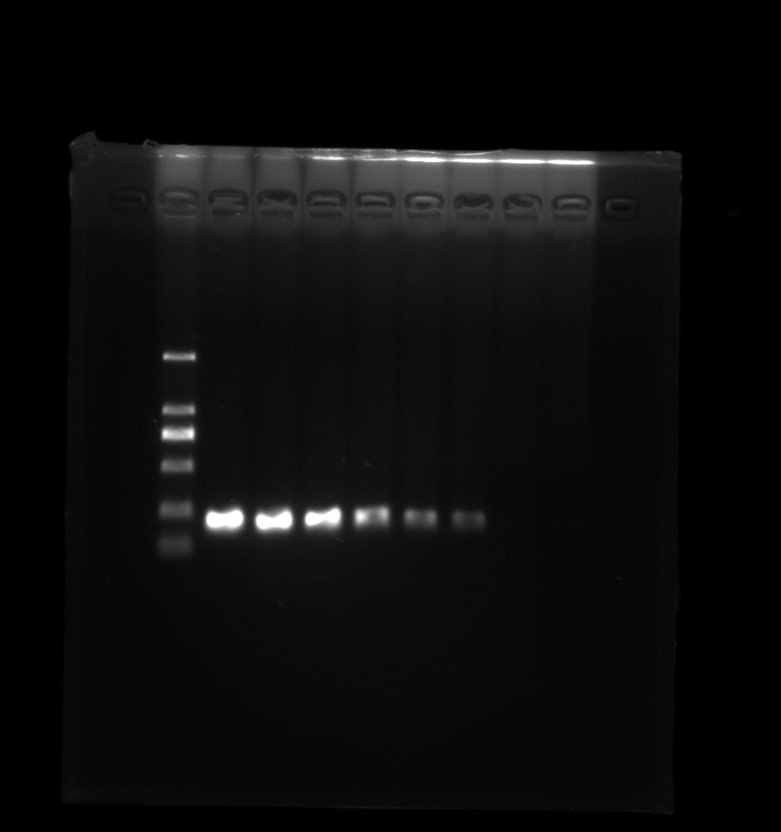

Supplement: Supplementary file 1 — Supplementary Material 1. [file 12866_2026_4881_MOESM1_ESM.zip › Supplementary materials - Original glue drawing/Original image(Fig. 3A).tif]

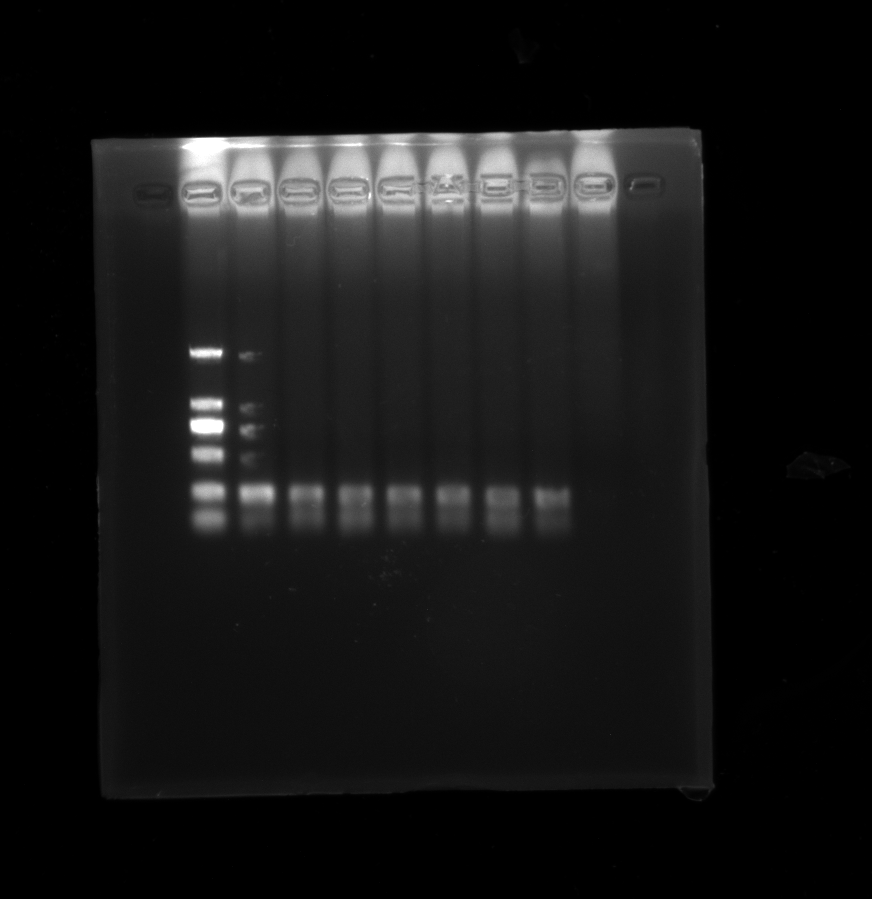

Supplement: Supplementary file 1 — Supplementary Material 1. [file 12866_2026_4881_MOESM1_ESM.zip › Supplementary materials - Original glue drawing/Original image(Fig. 3D).tif]

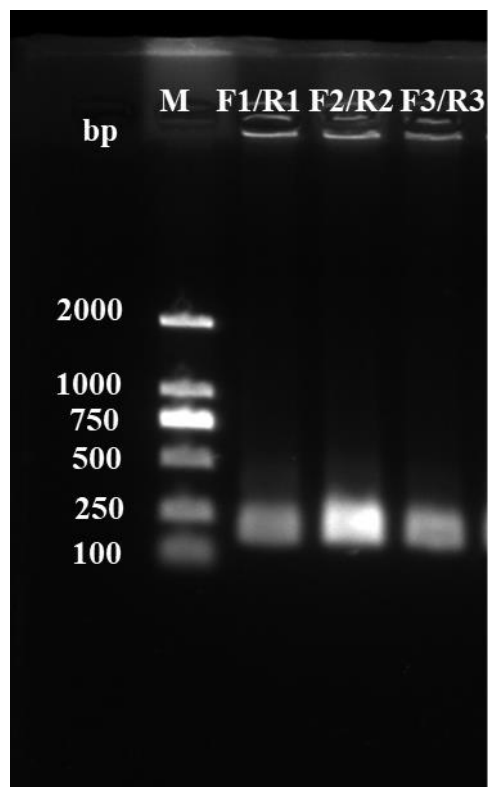

**Fig. 2A**

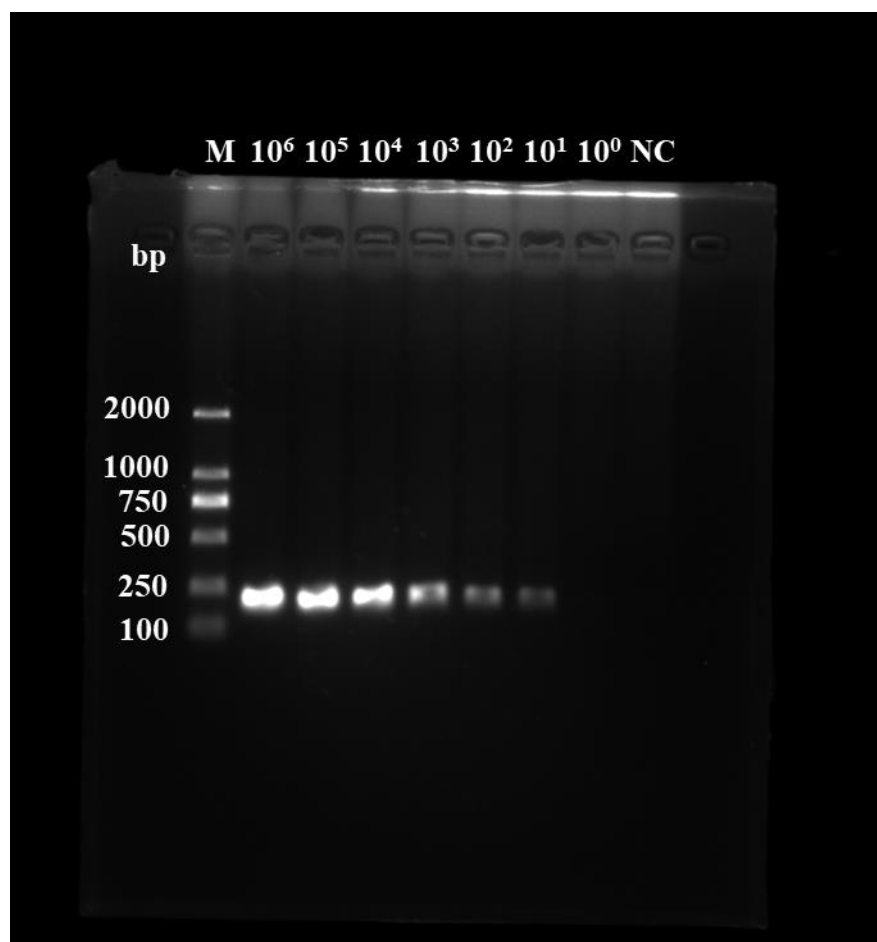

Fig. 3A

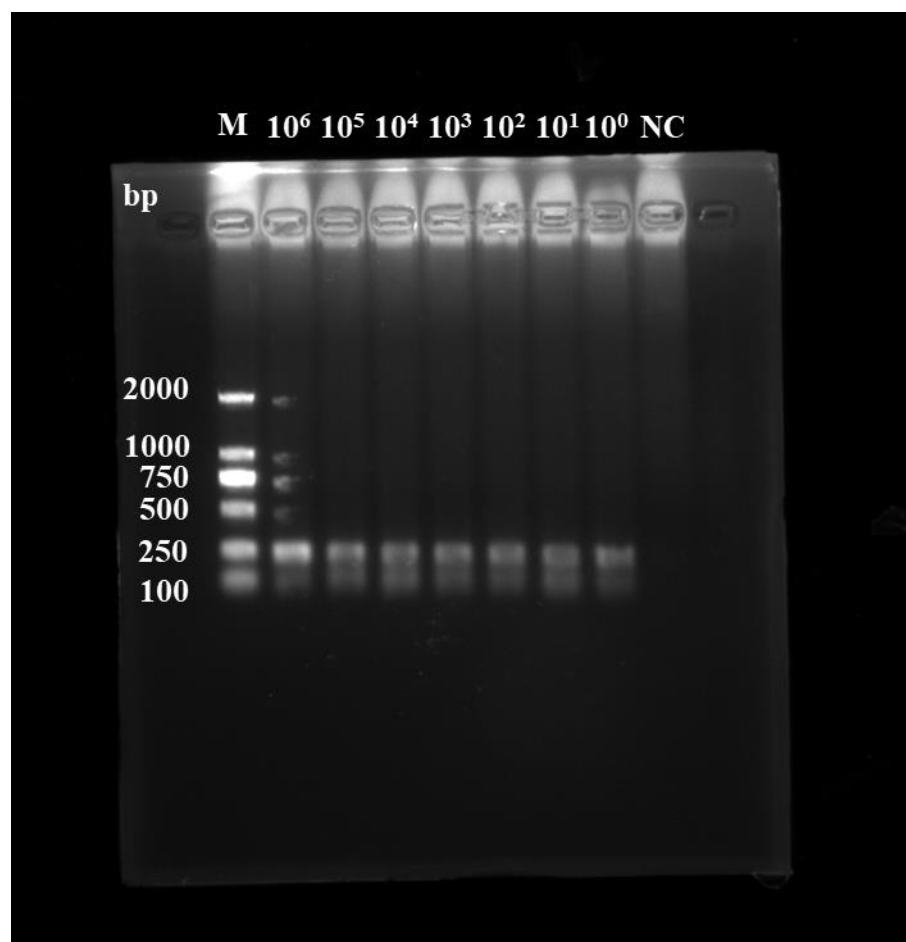

Fig. 3D

Supplement: Supplementary file 2 — Supplementary Material 2. [file 12866_2026_4881_MOESM2_ESM.pdf]
