# Supplementary material for: Development and application of a rapid detection system for Aspergillus fumigatus based on ERA/CRISPR-Cas12a
Source: BMC Microbiol. 2026 Mar 9;26:359. doi: 10.1186/s12866-026-04881-4 (PMC13085300; doi:10.1186/s12866-026-04881-4)
Supplement: Supplementary file 3 — Supplementary Material 3. [file 12866_2026_4881_MOESM3_ESM.docx]

**Materials and Methods**

**Materials and Instruments**

The A. fumigatus strain ATCC MYA-4609 utilized in this study was obtained from the American Type Culture Collection (ATCC). Conventional primers required for the experiments, along with the associated culture media reagents, were provided by Biotechnology (Shanghai) Co., Ltd. The P6 High-Fidelity Premix, LbCas12a enzyme, Cas12a High-Yield crRNA Synthesis and Purification Kit, modified ssDNA reporter probes (FAM and BHQ1 labels), and lateral flow test strips were sourced from Shanghai Tolo Harbor Biotechnology Co., Ltd. DNA purification kits were acquired from Tiangen Biotechnology (Beijing) Co., Ltd., while the ERA nucleic acid amplification kit was obtained from Suzhou Xianda Biotechnology Co., Ltd. The sputum extraction kit was purchased from Hangzhou Dilan Biotechnology Co., Ltd. A Roche LightCycler 96 system was employed for fluorescence quantitative PCR analysis, and the gel imaging equipment was sourced from Shanghai Qinxiang Scientific Instrument Co., Ltd. The fungal strains utilized in this research were preserved in our laboratory. Clinical *AF* sputum samples were supplied by Huaibei People's Hospital, following ethical approval by the hospital ethics committee (No. 2024-052).

**Cultivation of AF and Genome Extracti*on***

*AF* strains were cultivated on potato dextrose agar (PDA) plates at 30 °C for 4 days. Spores were then inoculated into 5 mL liquid PDA medium and incubated for 24 hours. Fungal hyphae and spores were collected, and genomic DNA was extracted following the fungal genome extraction protocol.

**PCR and ERA Amplification Systems**

The PCR reaction mixture (50 µL) contained 25 µL of P6 High-Fidelity Premix, 2 µL each of forward and reverse primers (10 µM), 2 µL genomic DNA template, and nuclease-free water. PCR amplification was conducted using an ABI PCR thermocycler. The cycling conditions included initial denaturation at 95 °C for 5 min, 35 cycles of 94 °C for 1 min, 55 °C for 1 min, 72 °C for 1 min, and final extension at 72 °C for 10 min.

The ERA amplification reaction (50 µL total) comprised 20 µL of dissolving reagent, 2.5 µL each of forward and reverse primers (10 µM), 2 µL of genomic DNA template, and 23.5 µL of nuclease-free water. This reaction mixture was added into tubes containing lyophilized reagents. Subsequently, 2 µL of activator was placed on the tube lid, the tube briefly centrifuged, and then incubated at 37 °C. Amplified products (2.5 µL each) from PCR and ERA reactions were subsequently analyzed by agarose gel electrophoresis.


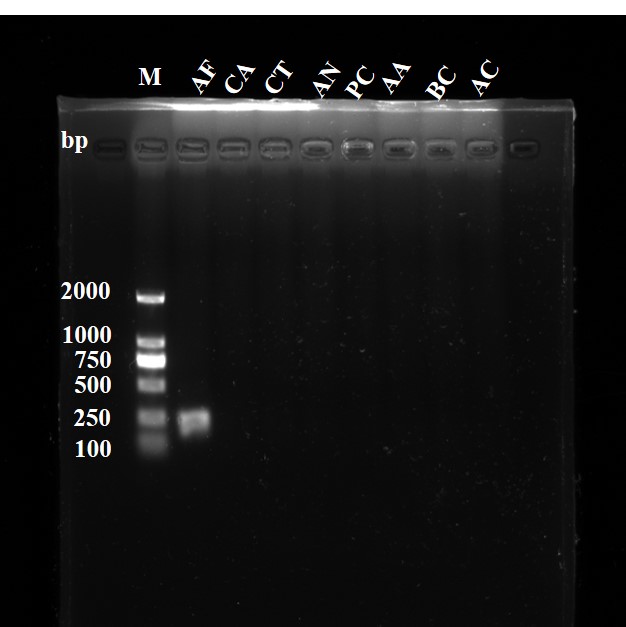


**Specific analysis agarose gel electrophoresis**


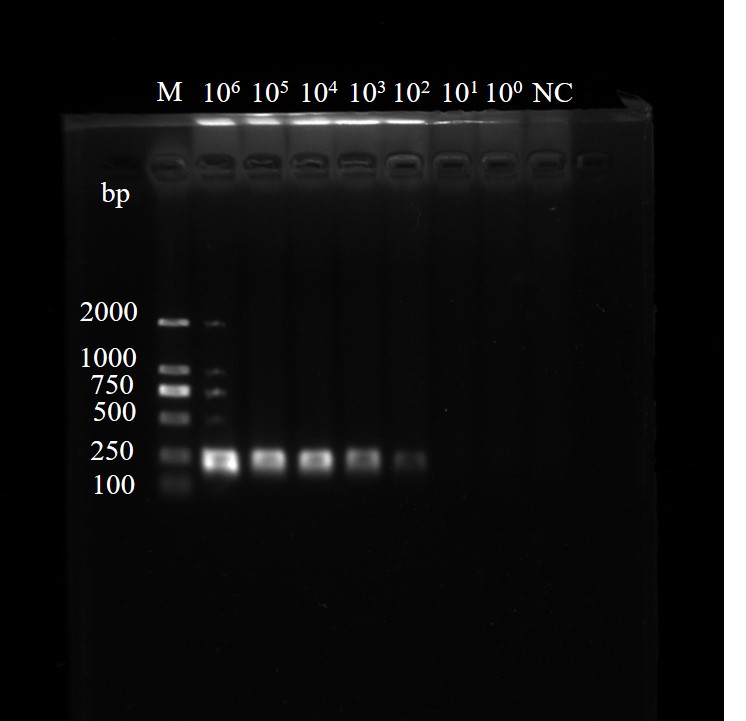


**Fig. 4A**
