# Supplementary material for: Development and application of a rapid detection system for Aspergillus fumigatus based on ERA/CRISPR-Cas12a
Source: BMC Microbiol. 2026 Mar 9;26:359. doi: 10.1186/s12866-026-04881-4 (PMC13085300; doi:10.1186/s12866-026-04881-4)
Supplement: Supplementary file 4 — Supplementary Material 4. [file 12866_2026_4881_MOESM4_ESM.docx]

**Materials and Methods**

**Primer specificity analysis**

To evaluate the detection spectrum and potential cross-reactivity of this assay against clinically relevant *Aspergillus* species, an in silico bioinformatic analysis was conducted using the designed ERA-F2/R2 primers. Primer sequences were aligned against the reference genomic of *Aspergillus clavatu*s (NW_001517097.1), *Aspergillus terreus* (NT_165936.1), and *Aspergillus lentulus* (NW_022983882.1), all retrieved from the NCBI Nucleotide database. Homologous regions corresponding to the target *anxC4* gene were identified across these genomes, and the number and positions of nucleotide mismatches within the primer-binding sites were systematically quantified. However, *Aspergillus flavus* was excluded from this alignment analysis due to the absence of a functionally annotated *anxC4* ortholog in current public genomic databases.

**Results**

To define the taxonomic scope of *Aspergillus* species detectable by this assay, we performed an in silico specificity analysis of the ERA-F2/R2 primer pair. The primers exhibited perfect complementarity to the target *anxC4* sequence of *Aspergillus fumigatus*. A complete match was also observed with the orthologous *anxC4* region in *Aspergillus lentulus*, supporting the potential utility of this assay for detecting members of the *Aspergillus fumigatus* species complex (Supplementary Fig. S1). In contrast, alignments with *Aspergillus clavatus* and *Aspergillus terreus* revealed 12 and 14 nucleotide mismatches (Supplementary Table S1)，thereby strongly limiting the likelihood of cross-amplification. Notably, no functionally annotated *anxC4* ortholog was identified for *Aspergillus flavus* in current NCBI genomic databases. Thus, experimental validation is required to determine its detectability and assess potential cross-reactivity.

**Supplementary Table S1.** Primer mismatch profiles relative to the *anxC4* genes of *A. clavatus*, *A. terreus*, and *A. lentulus*.

| Name | Mismatches (Forward) | Mismatches (Reverse) | | Total Mismatches |
| --- | --- | --- | --- | --- |
| *A. fumigatus* | 0 | 0 | 0 | |
| *A. clavatus* | 8 | 4 | 12 | |
| *A. terreus* | 8 | 6 | 14 | |
| *A. lentulus* | 0 | 0 | 0 | |


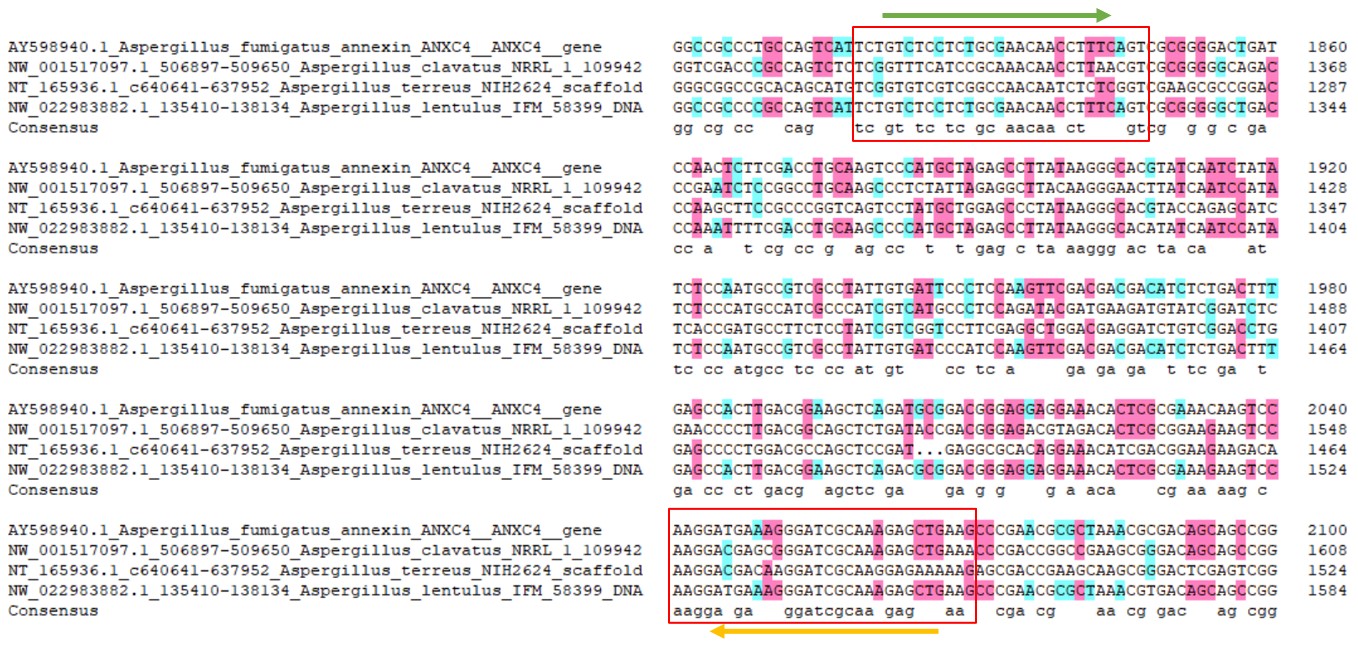


**Supplementary Fig. S1** Multiple sequence alignment of the *anxC4* gene from *Aspergillus fumigatus* and other Aspergillus species.
