# Supplementary material for: Development and application of a rapid detection system for Aspergillus fumigatus based on ERA/CRISPR-Cas12a
Source: BMC Microbiol. 2026 Mar 9;26:359. doi: 10.1186/s12866-026-04881-4 (PMC13085300; doi:10.1186/s12866-026-04881-4)
Supplement: Supplementary file 5 — Supplementary Material 5. [file 12866_2026_4881_MOESM5_ESM.docx]

**Supplementary Table S2.** Expanded panel of fungal strains used for inclusivity and exclusivity testing.

| Strain | Source of the strain | Strain quantity |
| --- | --- | --- |
| *Aspergillus fumigatus* | Isolated strain (Huaibei People's Hospital) | 13 |
| *Candida parapsilosis* | Isolated strain (Huaibei People's Hospital) | 1 |
| *Aspergillus terreus* | Isolated strain (Huaibei People's Hospital) | 1 |
| *Aspergillus flavus* | Isolated strain (Huaibei People's Hospital) | 1 |
| *Aspergillus versicolor* | Isolated strain (Huaibei People's Hospital) | 1 |
| *Aspergillus clavatus* | ATCC 9600 | 1 |
| *Cryptococcus neoformans* | Isolated strain (Huaibei People's Hospital) | 1 |
| *Rhizopus oryzae* | ATCC 56536 | 1 |
| *Fusarium oxysporum* | ATCC 48112 | 1 |


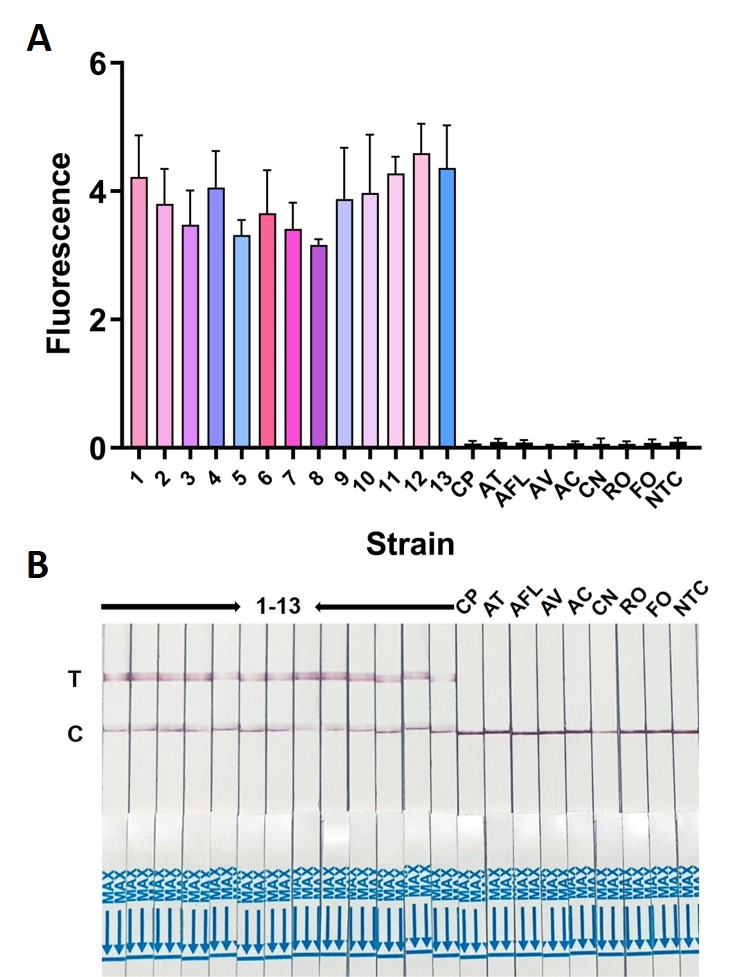


**Supplementary Fig. S2** The one-pot ERA-CRISPR/Cas12a detection system was used to validate analytical specificity using 13 clinical isolates of *Aspergillus fumigatus* and 8 additional fungal strains. (A) Fluorescence detection signals. (B) Lateral flow test strip results. Strains 1–13 are clinical isolates of *Aspergillus fumigatus*; the eight non-target fungal strains are abbreviated as follows: *Candida parapsilosis* (CP), *Aspergillus terreus* (AT), *Aspergillus flavus* (AFL), *Aspergillus versicolor* (AV), *Aspergillus clavatus* (AC), *Cryptococcus neoformans* (CN), *Rhizopus oryzae* (RO), and *Fusarium oxysporum* (FO)
